# P-916. Implementation of a Pharmacist-Led Anti-Methicillin-Resistant Staphylococcus aureus Therapy Bundle

**DOI:** 10.1093/ofid/ofaf695.1122

**Published:** 2026-01-11

**Authors:** Jeyma Fernandez, Alice M Landayan, Jorge Murillo, Grace Hoffman, Timothy Gauthier

**Affiliations:** South Miami Hospital, Miami, Florida; South Miami Hospital, Miami, Florida; South Miami Hospital, Baptist Health South Florida, Miami, Florida; South Miami Hospital, Miami, Florida; Baptist Health South Florida, Miami, FL

## Abstract

**Background:**

Pharmacist-led interventions can significantly improve antimicrobial use, optimize outcomes, and reduce antimicrobial resistance and adverse drug reactions. This initiative aimed to evaluate the impact of an anti-methicillin-resistant Staphylococcus aureus (MRSA) therapy bundle on pharmacist interventions and therapy use.

**Table 1.**
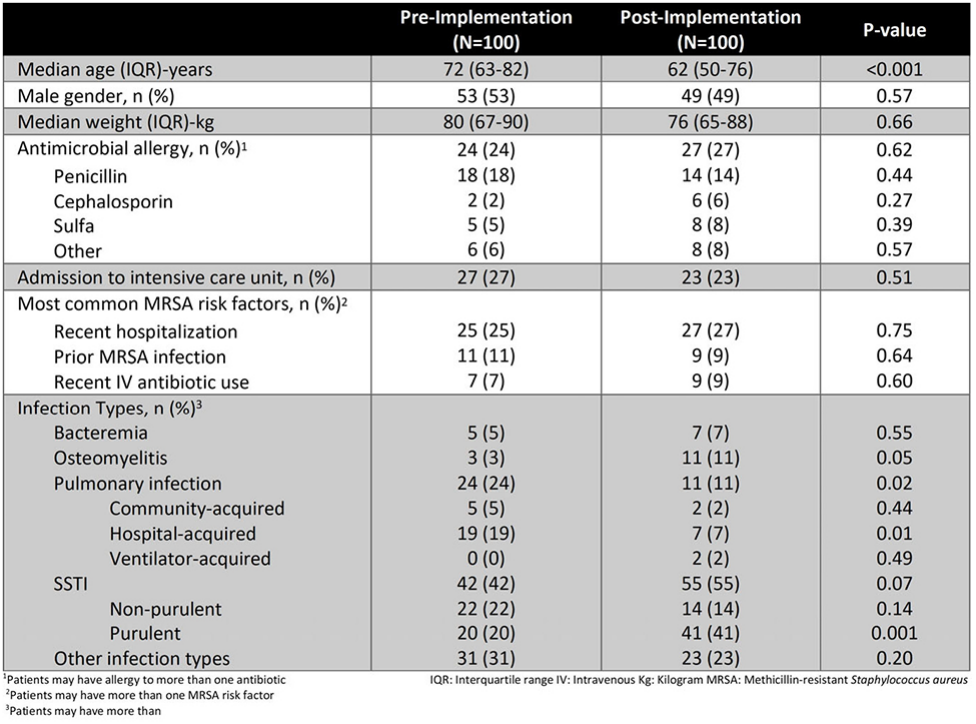
Baseline Characteristics

**Figure 1.**
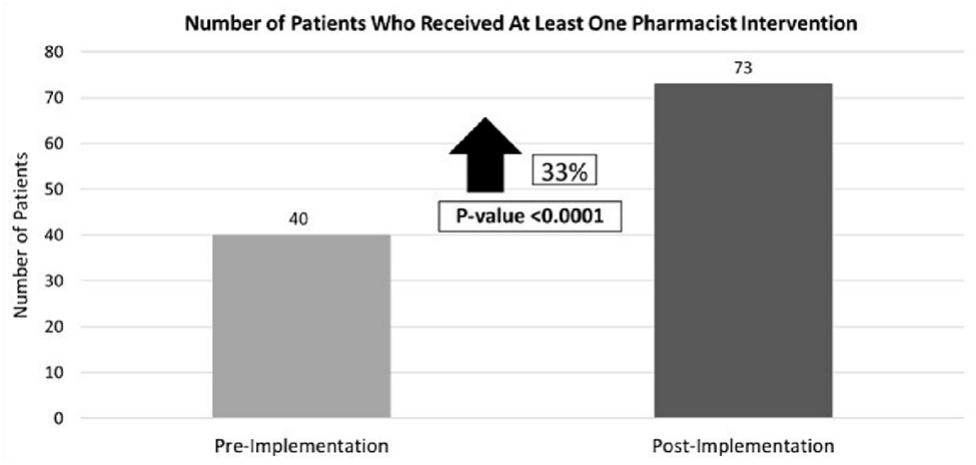
Primary Outcome

**Methods:**

This single-center retrospective project evaluated pharmacist impact before and after implementation of an anti-MRSA therapy bundle (January-March 2024 versus January-March 2025). The bundle included expanded VigiLanz® alerts, MRSA nasal polymerase chain reaction (PCR) use, an “Antibiotic Time-Out Tool: MRSA” document with pharmacist education, and daily VigiLanz® generated reports of active orders for daptomycin intravenous (IV), linezolid IV/by mouth (PO), and vancomycin IV. Hospitalized adult patients with orders for scheduled stated anti-MRSA agents were included. Patients were excluded if orders for “pre-op,” “on-call,” or “once” frequencies, indication of surgical or Group B Streptococcus prophylaxis, on chronic suppressive or prophylactic antimicrobial therapy, hospice and comfort measures only, incarcerated, or pregnant. The primary outcome was the number of patients who received at least one pharmacist intervention regarding daptomycin, linezolid, or vancomycin de-escalation, discontinuation, or optimization. Secondary outcomes included total number of pharmacist interventions, pharmacist intervention details, intervention acceptance rate, VigiLanz® adult hospital-wide inpatient days of therapy per 1000 patient days, hospital length of stay, incidence of adverse drug events, and 30-day mortality rate. This project was exempt from Institutional Review Board review.

**Table 2.**
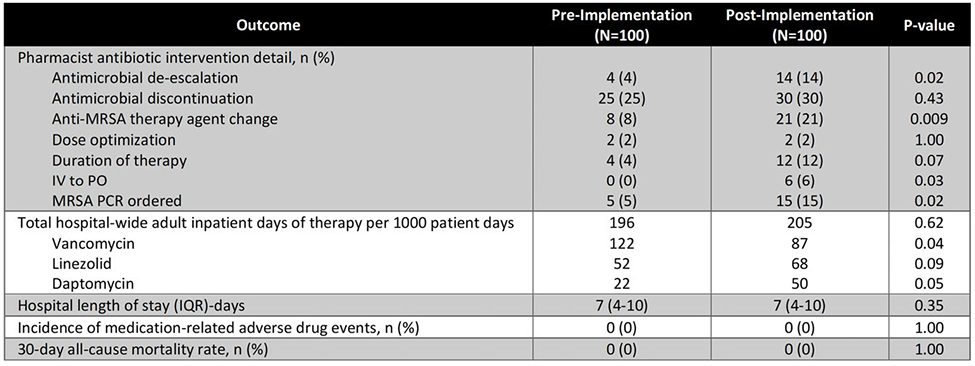
Secondary Outcomes

**Results:**

Baseline characteristics are listed in Table 1. Primary outcomes are detailed in Figure 1. The post-group had a 93% acceptance rate and 14% per-protocol completion. A total of 48 interventions were observed in the pre-group and 100 interventions in the post-group. Secondary outcomes are included in Table 2.

**Conclusion:**

A pharmacist-led anti-MRSA therapy bundle significantly increased interventions related to therapy de-escalation, discontinuation, and optimization, with high provider acceptance.

**Disclosures:**

All Authors: No reported disclosures

